# General and Device-Specific Reasons for ENDS Use: A Qualitative Study with Adult ENDS Users

**DOI:** 10.3390/ijerph19116822

**Published:** 2022-06-02

**Authors:** Mohammed M. Alqahtani, Zachary B. Massey, Robert T. Fairman, Victoria Churchill, David L. Ashley, Lucy Popova

**Affiliations:** 1 School of Health Professions, University of Alabama at Birmingham, Birmingham, AL 35294, USA; shwail.qahtani@hotmail.com; 2 Department of Respiratory Therapy, College of Applied Medical Sciences, King Saud bin Abdulaziz University for Health Sciences, Riyadh 14611, Saudi Arabia; 3 King Abdullah International Medical Research Center, Riyadh 11481, Saudi Arabia; 4 School of Journalism, University of Missouri, Columbia, MO 65211, USA; zbmassey@missouri.edu; 5 School of Public Health, Georgia State University, Atlanta, GA 30302, USA; rfairman1@student.gsu.edu (R.T.F.); vchurchill1@student.gsu.edu (V.C.); dashley4@gsu.edu (D.L.A.)

**Keywords:** electronic cigarettes, reasons for use, device characteristics, vaping

## Abstract

Background: A scientific consensus on the public health impact of electronic nicotine delivery systems (ENDS) remains elusive. This is partly due to the wide variation in product characteristics often lumped together under one category. Research is needed to better understand what ENDS device type characteristics motivate their use by adults. Methods: Nine focus groups of 32 current ENDS users who were 18+ years old, had used ENDS in the previous 30 days, and had been using ENDS for more than two months were held either in person or online between February and June 2020. Results: Participants’ reasons for their choice of ENDS characteristics included both general, applying to all ENDS products, and specific, relating to particular ENDS devices. Health benefits and the lack of offensive odor were commonly identified as important reasons for using ENDS in general. Flavor and product discreteness were both general and device-specific determinants of ENDS use. Conversely, nicotine delivery, cloud size, battery properties, aesthetics, ease of use, and cost were device-specific drivers of participants’ choice. Conclusions: The reasons that adults choose to use ENDS are complex and sometimes related to both ENDS as a category and as specific ENDS product types. Regulations and public communication campaigns should reflect their ultimate objective and consider both general and specific motivations when attempting to achieve public health objectives.

## 1. Introduction

In the United States, the use of electronic nicotine delivery systems (ENDS) or electronic cigarettes (e-cigarettes) has increased dramatically since 2010 [1,2,3]. Approximately 8.1 million adults in the United States were using ENDS in 2018 [4]. ENDS typically comprise battery-powered coils that heat and aerosolize e-liquids containing propylene glycol and/or vegetable glycerin, nicotine, and flavors [4,5,6,7]. ENDS have a variety of physical designs and features. The evolution of ENDS has been described in terms of generations: the first generation products included *cigalikes*, closely resembling cigarettes in appearance, the second generation *vape pens*, the third generation *mods*, which allowed more user adjustments, and the fourth generation *pod-mods* (such as Juul or Sourin) [8]. However, the distinction between generations has not been clear-cut, and new products sometimes combine features of different generations [8].

Reasons for ENDS use among adults vary and have been well studied. For example, avoiding smoking restrictions [9], reducing the harmful impact of smoking [10] while mimicking the sensation of smoking a combustible cigarette [11,12,13], weight control [14,15], flavors [14], the convenience of product use [16], the novelty of the devices, and smoking cessation [17], and social acceptability [18] were named as reasons for ENDS use. However, most of the studies on the reasons for ENDS use asked about ENDS as a general category of tobacco products without differentiating between specific devices or features. In addition, a sizeable body of research has examined preferences for specific device characteristics, such as flavors, nicotine strength, or device type [19,20,21]. However, the two approaches (reasons for ENDS use and preferences for ENDS characteristics) have generally remained separate. To our knowledge, no studies have examined how the same ENDS users explain their reasons for using ENDS through both general category and characteristic-specific lenses. 

Given the variety of devices and features, it is likely that some reasons for product choice only apply to some devices or features, and other reasons might be widely applicable to ENDS as a class of products. For example, users might believe that some types of devices are better at helping them quit smoking, but they might think that any ENDS would be helpful in weight control. Currently, we do not have this information, but it is important to inform potential product regulations. For example, to minimize the appeal of products to non-smokers, the US Food and Drug Administration (FDA) might only allow closed ENDS to be sold (similar to the closed ENDS devices with tobacco-flavored cartridges such as Vuse Solo that the FDA authorized in October 2021 [22] and Logic Vapeleaf, Logic Pro, and Logic Power that were granted marketing orders in March 2022) [23]. However, this could affect the use of these products by smokers trying to quit cigarettes. How these decisions limiting the range of available product characteristics may impact the current use of these products is an important consideration to any regulatory action. For instance, removing all products with a particular attractive characteristic could encourage users to abandon ENDS because the limited device characteristics no longer meet their reasons for use. On the other hand, users may readily switch to other ENDS products if the market availability contracts, but other products are available that address the primary appeal to a general categorical reason for use. Our investigation aimed to explore this issue. 

## 2. Materials and Methods

These data come from a focus group study conducted as part of a larger investigation on ENDS modifications. The main focus of the study was on the types of modifications and reasons for them (reported in a different paper [24]), with a secondary focus on the reasons for use for different devices. The reasons for use came up throughout the focus groups discussions. Even though we did not explicitly ask about reasons for use of ENDS in general and for specific devices, the responses naturally reflected this division, which we further elucidated in the data coding and analysis. The reasons for using the ENDS were classified into two categories: general reasons for using ENDS and reasons based on specific ENDS devices or features. 

We used a qualitative description approach (QD) to conduct focus groups and analyze the data. In QD, data analysis strives to gain a detailed and straightforward description that stays close to the data, with results reported in the original language of the participants [25,26]. Compared to other types of qualitative research, QD emphasizes the discovery of truly illustrative information, particularly from a topic that is not well understood [27]. Therefore, rather than prescribing a specific lens or theory in which to interpret the qualitative data, the authors aimed to gain insights solely through the words of the participants. A qualitative description approach is recommended for focus group studies [26], where participants speak openly and freely, giving opinions on many topics [28]. This paper is organized according to the Standards of Reporting Qualitative Research [29].

### 2.1. Research Team and Reflexivity

The research company John Snow Inc. (JSI, Boston, MA, USA) recruited participants and ran focus groups. The study lead was a Licensed Social Worker with a Master’s in Public Health. Another JSI employee assisted the study lead. Together, the JSI team recruited and moderated all focus groups. Outside JSI staff, no members of the research team interacted with participants.

### 2.2. Participants and Procedures

Purposeful sampling [30] was used to select current ENDS users (18+, used ENDS in the past 30 days, and had been using for more than 2 months) who were likely to be knowledgeable about ENDS use behavior. Participants were recruited online (Facebook ads, Craigslist postings) and offline (e.g., flyers in the city of Atlanta for the initial recruitment in February 2020). Interested individuals completed online screening about demographics and tobacco use, and those eligible were contacted to participate in focus groups.

Thirty-two adult ENDS users participated in nine focus groups from February to June 2020. The first three groups were in-person and held in Atlanta, GA, USA. After the onset of COVID-19 in March 2020, the remaining six groups were conducted using videoconferencing software with participants from across the country. The two JSI staff moderated focus groups using open-ended questions with a structured interview guide [26]. The moderator guide was developed based on our previous study (interviews with adult ENDS users [13], extant literature, and consensus by the research team comprising interdisciplinary experts in ENDS use behavior). Participants were asked what devices they used, what they liked about their devices, how they used them, and how they learned to use them. We also asked about modifications to the devices or e-liquids, and these results have been published elsewhere [24]. Focus groups lasted 38 to 81 min (median 71 min). Group size ranged between one (in the last in-person focus group due to no-shows at the beginning of COVID-19) and six (median = 3). Focus groups were audio-recorded. Each participant received USD 50 compensation. The Georgia State University Institutional Review Board approved the study. All participants provided electronic and verbal consent.

### 2.3. Data Analysis

Focus group discussions were transcribed and anonymized by JSI staff before dissemination to the research team. Transcripts were analyzed using a qualitative description approach [26,28]. Specifically, Z.B.M. read the transcripts and developed an initial codebook based on themes that closely followed participants’ answers about ENDS use behavior. Next, the research team met to discuss coding themes and refine codes. R.T.F. and V.C. independently coded two focus group transcripts. All coding discrepancies were discussed with Z.B.M. and resolved, and the codebook was updated and revised. R.T.F. and V.C. coded the remaining transcripts in NVivo 12 [31]. All authors reviewed coded transcripts, wrote memos summarizing the results of each code, and then met to discuss those results. M.M.A. synthesized findings from the memos. 

## 3. Results

### 3.1. Sample Characteristics

Table 1 displays participants’ sociodemographic and tobacco use characteristics. Participants discussed various reasons for using ENDS. In the following sections, we list the reasons and address whether each was discussed applying to ENDS in general, applying only to specific devices/features, or both. Health benefits and the lack of offensive odor were discussed exclusively in general ENDS terms, without referring to specific devices or features. Flavor and discreteness were mentioned in terms of both general and device-specific reasons. In contrast, nicotine delivery, cloud size, battery, aesthetics, ease of use, and cost were discussed primarily concerning specific devices and features. Table 2 provides example quotes for each category of reasons.

### 3.2. Reasons for Use General to ENDS

#### 3.2.1. Health Benefits

Discussion about the health benefits of ENDS was primarily framed in general terms rather than specific devices. In almost all focus groups, participants described ENDS as healthier than cigarettes and frequently cited health concerns about smoking as the original reason they started using ENDS. Some participants mentioned their general beliefs of ENDS being healthier because they do not have “10,000 chemicals” like cigarettes, while others recounted their own experience feeling better after switching to ENDS. One of the focus groups extensively discussed the state of the science regarding the safety and health effects of ENDS and clarified that health issues related to ENDS use (i.e., e-cigarette or vaping use-associated lung injury or EVALI) were caused by “off-the-street vitamin E acetate” (32, M). The only mention of ENDS features related to health dealt with “safety features” of devices, including “being able to lock the device, so it doesn’t explode”. 

#### 3.2.2. Lack of Offensive Odor

Another perceived benefits of ENDS—the lack of offensive odor—was discussed in terms of general ENDS use rather than device-specific reasons. Participants mentioned using ENDS to smell better—either to avoid the noxious odor of cigarettes or to smell like flavored e-liquids. As one participant put it, ENDS users “want to sometimes not offend the people around them with the old nicotine smell. So, they will have those flavorful ones, to kind of give it a better aroma around the room or wherever they’re at” (28, M). For one participant, “the only reason why I switched over to e-cigarette is because of the ashes and the cigarette smell” (32, F). The lack of unpleasant smell allowed some participants to use ENDS at home without upsetting their families. Participants did not differentiate among devices and features based on the odor. 

### 3.3. Mixed Reasons for Use (Both General to ENDS and Specific to Devices/Features)

#### 3.3.1. Flavor

Flavor was discussed extensively as a reason for using ENDS, predominantly in terms of general ENDS use, but with some discussion of specific devices. When participants referred to flavors of ENDS in general, they discussed the appeal of flavors, such as menthol, citrus, vanilla, cherry, watermelon, peach, and strawberry. One participant described how the sweetness of the flavor is linked to the ENDS enjoyment and the frequency of use: “If it’s sweeter, it’s going to taste better to me and probably a majority of people, and you would use it more often because of that” (23, M).

In terms of specific devices, participants focused on device attributes that enabled flavor switching. For example, some mentioned that refillable ENDS allow changing the flavor easily: “I personally like refillable because I like to try different flavors; […] with refillable, you’ll be able to access a lot of more different types of flavors versus using disposable ones” (24, M). Participants that used tank devices also appreciated their ability to switch flavors. A participant using a SMOK device commented that “you can customize the flavor that you want, and you can customize the strength on the fly that you would like” (36, M). In contrast, some participants mentioned that pod-based ENDS (such as Juul and Myblu) and disposable devices are limited in terms of flavor: “they’re restricted to their own brand’s flavor” (36, M), and at least one participant said that “it kind of discourages me from using them too much because they don’t offer a lot of flavors” (40, F).

#### 3.3.2. Discreetness

Discreetness of ENDS was discussed in both general and device-specific terms. For general reasons, multiple participants mentioned ENDS enabling tobacco use in situations where smoking would bother others or where others would judge the smokers. For instance, participants discussed using ENDS to avoid attention from other people (“nobody really has to know”) (44, F), or vaping in a way that does not bother people the same way cigarettes would.

In terms of specific devices, participants repeatedly mentioned smaller devices that are easier to conceal. For example, one participant appreciated “The Juul, I like that it’s flat and it’s skinny and you can sneak it places, so you can bring it places you’re not supposed to be, and use it” (30, F). Another mentioned that larger devices are more difficult to conceal, providing further evidence of a preference for small ENDS devices when wanting to remain discrete: “So having a big bulky mod is different than just having something that you can stick in your pocket” (32, F). Thus, focus group discussion revealed that many participants used ENDS based on the ability to mask or hide the device and remain discrete regarding use. 

### 3.4. Reasons Specific to Devices/Features

#### 3.4.1. Nicotine Delivery

Multiple participants said that the nicotine level is critically important, describing this characteristic as a general issue. However, when participants explained how they achieve the preferred nicotine level, the discussion of reasons switched to specific devices and features. In such cases, participants indicated they chose devices to obtain a subjective—but specific—perceived effect of nicotine. For instance, some participants described changes in nicotine levels over time. When ENDS devices first hit the market, available nicotine levels were low, and as one participant indicated, they “couldn’t feel it, they couldn’t taste it” (29, M). However, then “they [ENDS devices] started changing, they started getting more stronger” (28, M). Some participants discussed shifting their preference for nicotine levels over time—starting stronger and tapering down by using different devices. Participants also mentioned a preference for “stronger” or “bigger” nicotine hits and “weaker” or “smaller” hits depending on the time of the day or setting and compared using ENDS to smoking cannabis and drinking different kinds of alcohol. Participants named specific device types and brands that provided higher nicotine levels, with multiple participants referring to Blu and some discussing larger devices, such as SMOK mods. However, while nicotine discussions mentioned specific device types, perceptions about nicotine concentration and effect were mainly subjective and not clearly linked to device labels or listed concentrations. Participants described using certain devices to obtain specific and subjective effects perceived to be related to nicotine delivery. 

#### 3.4.2. Cloud Size

Discussions of clouds were mostly device specific. There were some general observations, such as that bigger clouds correspond to higher nicotine delivery and that the importance of cloud size diminished with time. However, despite indications that cloud tricks may be waning in popularity, participants repeatedly mentioned that controlling the cloud size (either to be very large or very small) was an important characteristic of ENDS and the reason to select specific devices. For a few participants, discreteness related to the vapor produced by the devices was important, “If anything, I don’t want people to see me blowing huge clouds” (23, M). For others, bigger clouds were a must for nicotine delivery and entertainment, and bigger mods were able to provide a “satisfying” and “fun” experience better than “Juul stuff” and “little pod mods.”

#### 3.4.3. Battery

Battery durability was predominantly discussed as device-specific. In terms of the general reasons for use, most of the participants mentioned that battery life and longevity of the ENDS were essential for them. Regarding specific devices and features, three features were mentioned: how fast the battery charges (e.g., Blu “charges really, really fast. It dies quickly because it’s so tiny, but it charges really quick as well”) (28, M); removable batteries (e.g., “you can get a separate charger and charge multiple batteries at the same time. […] Then, I will just carry a backup battery”) (35, F); and having a battery life indicator. Participants also mentioned that type and performance of the battery dictates how people use the device when the battery runs low (e.g., “you better conserve like you conserve to cut down on a pack of cigarettes”) (36, M).

#### 3.4.4. Aesthetics

Participants discussed aesthetics as the reason for using specific devices. Several participants indicated that different ENDS design features, such as color, size, weight, and appearance, were appealing. For instance, one participant admired mods because of the availability to obtain different colors and designs. “I know that my mod has different colors and different types of designs that you can pick from. So yeah, I like to kind of color match mine” (21, F). Other participants mentioned that they like the resemblance between ENDS devices and cigarettes. For example, “the main reason why I chose Blu is because it still has that cigarette feel, like when you’re holding it,” (32, F) and “Vuse, it feels more like a cigarette and it has the same feeling of a cigarette” (32, F).

#### 3.4.5. Ease of Use

Ease of ENDS use was primarily mentioned in terms of device-specific reasons. One was device portability. A participant who uses Blu and Juul commented that they were “lightweight and easy to carry along. I could take [them] anywhere with me” (40, F). Another was the ease of using and maintaining the device, where participants were split, with some preferring smaller closed systems (e.g., pods) and others favoring open systems (e.g., box mods). Pods were preferred by participants who wanted to change the pods out quickly instead of refilling tanks. In contrast, tank and box mods users mentioned that they did not have to fill them up as much and did not need to worry about finishing out or switching the cartridges like they would with pods. Filling up tanks was described as easy, and, since it has been a part of their experience for a long time, maintaining the tank devices was “almost second nature,” such as: “You can do it on autopilot and not even think about it. It’s like when you have your coffee in the morning. You just do it” (26, M).

#### 3.4.6. Cost

Participants discussed cost mostly in terms of specific devices, differentiating between cheaper and more expensive brands. The cost, to some extent, determined the use of the device: participants worried about losing expensive devices, so they only used them at home and used cheaper devices outside the home. A few participants were willing to pay premium prices for specific products: “your final product is only going to be as good as what you put in it. So, I don’t mind paying a little bit extra for a [good] flavor” (42, F). However, most were interested in finding more affordable products. Participants talked about refillable box mods being cheaper than closed pods and one person mentioned “Vuse Alto” as being particularly inexpensive (Table 2).

## 4. Discussion

To our knowledge, this is the first qualitative study analyzing reasons for ENDS use contrasting general and device-specific reasons. In the focus groups, current ENDS users reported various reasons for using ENDS, classified into two categories: reasons general to ENDS and reasons specific to ENDS devices and features. Health benefits and lack of offensive odor were reasons discussed predominantly in terms of ENDS in general; flavor and discreteness had both general and device-specific reasons, whereas nicotine delivery, cloud size, battery, aesthetics, ease of use, and cost were mostly described as device-specific. Our study provides preliminary answers on the possible effects of future regulations and restrictions on ENDS use in general and specific devices or features, particularly regarding flavors, nicotine levels, or modifiability of devices.

Two reasons—health benefits of ENDS and their lack of offensive odor—were discussed predominantly in general terms. Health benefits of ENDS (or reduced harm of ENDS compared to cigarettes) have been frequently listed as one of the main reasons for ENDS use [18]. In our study, participants did not differentiate between ENDS devices or features when describing how they started or continued using ENDS for health reasons and talked about health benefits in terms of ENDS in general. Similarly, the lack of offensive odor was viewed as a general feature of all ENDS compared to cigarettes and not device-specific. This indicates that those using ENDS predominantly for health reasons or to avoid offending others might switch to other ENDS products if their specific devices are no longer available due to regulatory decisions, since those benefits are perceived to be common to all ENDS, regardless of the device type.

However, consumers use ENDS for many other reasons [18], most of which were discussed at least partially in terms of specific devices or device features. Two reasons—flavor and discreetness—were discussed in terms of both ENDS-general and device-specific features. Flavor was repeatedly mentioned as important to participants, which corresponds with previous findings that flavor is one of the motives for using ENDS [32,33]. The role of specific devices in our study became evident when participants described how they could switch between the flavors with some devices (e.g., refillables or tanks), allowing more flexibility than devices that come with prefilled pods. Regulatory action that removes specific ENDS devices that allow more flavor flexibility may discourage the use of ENDS as an alternative to smoking for some ENDS users who associate their specific product with flavor choice. Other ENDS users who are attracted because of the use of flavors in general may be able to switch to other products if their current product of choice is removed.

Nicotine delivery was widely discussed among the participants as one of the primary factors for using ENDS. Participants differentiated between devices able to consistently provide the desired level of nicotine and those that did not, such as earlier generations of ENDS. Moreover, our results showed that participants tended to rely on subjective perceived effects of nicotine delivery instead of label concentrations for nicotine. Nicotine is the principal driver of addictive tobacco use [34], and the ability of ENDS to provide the same satisfaction (i.e., nicotine delivery) as cigarettes was a determining factor on whether a smoker rejected ENDS [35]. These results provide important descriptive findings about how the perceived effects of nicotine and device characteristics influence consumers, information that informs regulatory decision making about ENDS. Regulatory action that restricts nicotine levels may result in some users rejecting ENDS if this is a critical reason for their product use and the products that remain available are not what they need to address their dependence.

As the FDA moves forward with regulatory actions on ENDS, decisions will be prioritized regarding the available scientific evidence and the likely impact on public health. Regulatory actions that reduce the diversity of ENDS products may have both positive and negative outcomes due to the use of ENDS both as a product that leads to or maintains nicotine dependence and serves as a pathway out of combusted cigarette smoking. For example, our research has shown that ENDS flavors motivate product use both in general and related to specific products. If all non-tobacco flavored ENDS are removed from the market, this is likely to substantially reduce the use of ENDS broadly due to the general appeal of flavors to users. Conversely, a more targeted flavor ban, for example, not allowing refillable flavor pods but permitting a limited number of flavors in closed ENDS devices, may reduce use of ENDS for certain groups who consider the opportunity to quickly switch between flavors important (e.g., youth), while still allowing options for those who are willing to accept less variation in their choice of flavors. The FDA should consider both the intended results of their actions and the unintended consequences to effectively promote public health. 

In considering what ENDS users would do if their preferred devices or features are no longer available, it is important to prevent ENDS users from returning to smoking combusted cigarettes. If the objective of regulatory actions is to continue availability of ENDS that will encourage smokers to switch completely from smoking, the products that remain on the market must appeal to smokers adequately to encourage that transition. Thus, the availability of some products with those characteristics that appeal to ENDS users in general is important for the continued impact of ENDS as a disruptive technology for smoking. In examining the reasons for use through this lens, it seems that those who predominantly use ENDS for health reasons, to quit smoking, or not to offend others may not return to smoking if some ENDS are taken off the market, but others remain. However, those who use ENDS for nicotine delivery and cost may quit ENDS use and possibly return to smoking if the specific products that motivate their use are removed. 

### 4.1. Limitations

The generalizability of our results is limited due to the small, non-representative sample of participants. Focus groups were conducted during the onset and continued COVID-19 pandemic in the United States. Most groups used videoconferencing technology, which may have affected the conversational norms of face-to-face discussions, such as maintaining eye contact or not talking over other participants. In addition, using online screening and videoconferencing software could have led to selection bias based on internet access. Additionally, we did not ask each participant what type of e-cigarettes they used or the number of device types they tried. While some participants discussed this in the focus groups, we do not have systematic information on this for all participants.

### 4.2. Future Research

Many studies on the reasons for ENDS use exist, but they have not been summarized in a meta-analysis or a review that would allow us to rank the most important reasons for ENDS use. Studies are lacking on what ENDS users would do if certain device features were no longer available, except for studies examining hypothetical reactions to flavor bans [35]. Research is needed to determine how other regulations (such as taking open systems off the market or increasing cost) would affect ENDS consumers with different reasons for use. Our study examined how these various reasons were discussed organically; more systematic studies should examine the interplay between ENDS use and reactions to potential restrictions of ENDS devices or features. 

## 5. Conclusions

Despite a large body of research about the general reasons for using ENDS products and studies examining preferences for specific devices and features, there is a gap in our understanding of to what extent the reasons for ENDS use are device-specific instead of general. We found that health reasons and the lack of offensive odor were the reasons that applied to ENDS broadly, that flavors and discreteness were discussed in terms of both general and device specific reasons, but that nicotine delivery, cloud size, aesthetics, ease of use, and cost were device specific features. It is likely that if the availability of specific ENDS characteristics were restricted, those who use ENDS for health reasons or to avoid offending others might switch to remaining authorized devices. However, for those for whom specific ENDS characteristics, such as cloud size, ease of use, battery durability, and nicotine delivery, are critical, restrictions on these specific features might make it less likely that users will switch to different ENDS products that do not include these features. Precautions also need to be taken that ENDS restrictions do not make cigarettes more appealing, which can happen with insufficient nicotine delivery [34] or increased cost [36,37,38]. The FDA and other regulators should consider this when conducting product authorization assessments and developing product standards.

## Figures and Tables

**Table 1:** Characteristics of Focus Groups Participants.

Variable	*n* (%)
**Sex**	
Male	17 (53.1)
Female	15 (46.9)
**Age**	
18–29	18 (56.3)
30–44	13 (40.6)
45–59	1 (3.1)
**Race**	
Asian	3 (9.4)
Black or African American	8 (25.0)
White	21 (65.6)
**Ethnicity**	
Spanish, Hispanic, or Latinx	4 (12.5)
**Tobacco use status**	
Never smoker, current ENDS user ^a^	7 (21.9)
Former smoker, current ENDS user ^b^	7 (21.9)
Current smoker, current ENDS user ^c^	18 (56.3)
**Vaping frequency** ^d^	
Every day	23 (71.9)
Some days	9 (28.1)
**Duration of ENDS Use** ^e^	
1 year to 5 years	17 (53.1)
6 years to 15 years	14 (43.8)

^a^ Among never smokers, 6 reported having never smoked tobacco cigarettes and 1 reported having ever smoked tobacco cigarettes but not having smoked 100 cigarettes in their lifetime. ^b^ Former smokers had smoked over 100 cigarettes in their lifetime but were not currently smoking (Selecting “not at all” in response to “Do you now smoke cigarettes every day, some days, or not at all?”). ^c^ Current smokers had smoked over 100 cigarettes in their lifetime and were currently smoking “every day” or some days”. ^d^ Measured as “Do you now use electronic vapor products every day, some days, rarely, or not at all?” No participant selected “rarely” or “not at all”. ^e^ Calculation for duration of use was age minus the age first used ENDS (M = 5.6 years, SD = 3.18, Median = 5.0, Range: 1–15 years). One entry treated as missing data because of invalid entry.

**Table 2:** Reasons for using ENDS in general and specific devices/features.

Reason	General to ENDS ^a^	Device-Specific ^b^
**Predominantly general reasons**		
**Health benefits**	“Vaping is a lot more safer now, to a certain degree, than regular cigarettes” (38, M). “I know a few of my friends switched from cigarettes to using e-cigarettes or vapes. Probably for health reasons, because whatever you want to believe, smoking cigarettes is worse than using a vape” (23, M). “There’s definitely a case to be made that, yeah, inhaling anything is inherently detrimental to your health. You know, oxygen can be bad if you get too much of it. But is this currently a safer alternative than the stuff that people have been smoking since they invented times, yeah, absolutely” (28, M).	“I think what makes it safer altogether too, and one reason I buy SMOKs because not all mods have it is, I can lock this. So, if it goes off in my pocket, it’s not going to explode in my pocket. Burn everything” (25, M).
**Lack of offensive odor**	“The only reason why I switched over to e-cigarette is because of the ashes and the cigarette smell” (32, F). “No one wants to walk around smelling like an ashtray” (32, F). “But the newer one, I feel like those flavors just, it gives everybody a different accommodation to what they use. And also, they want to sometimes not offend the people around them with the old nicotine smell. So, they will have those flavorful ones, to kind of give it a better aroma around the room or wherever they’re at, to vape” (28, M). “It doesn’t have any scent to smell up the house, because when I used to smoke my husband don’t smoke, so it would stink on the furniture and things of that nature, so I could do it when I want” (52, F).	-
**Mixed general and device-specific reasons**		
**Flavor**	“I’m like, ‘If they were to ban all flavors and only have like a tobacco, I would probably stop’” (21, F). “The biggest reason, the biggest issue that I switched from cigarettes to vape was because of flavor” (36, M). “Yeah, I like that too, having the freedom to choose. If I’m in a mood for something else, I can just get a different, swap it out. Super easy” (23, M).	“I personally like refillables because I like to try different flavors. I feel like with refillables, you’ll be able to access a lot of more different types of flavors versus using disposable ones” (24, M).
**Discreteness**	“I remember whenever people first started vaping that was a big thing, the tricks and stuff. But I think most people now are more worried about being discrete and not being judged” (28, M). “Instead of actually worrying about going outside and offending people with a strong cigarette, you can have an e-cigarette somewhere and the smoke kind of just aspirates a lot more quicker than regular cigarettes” (28, M).	“The Juul, I like that it’s flat and it’s skinny and you can sneak it places, so you can bring it places you’re not supposed to be and use it” (30, F).
**Predominantly device-specific reasons**		
**Nicotine delivery**	“I would pick higher when I first started, and then I slowly was like, ‘I don’t need this amount. It’s fine. I don’t need to go that crazy’” (21, F).	“I just get […] the smaller ones and I just go slowly throughout the day […]. It’s kind of like to me, it’s between beer and alcohol. Beer, you drink a couple of beers, it gets you a slower buzz, but with liquor, you’re going to go straight to the top. That’s kind of like how I compare vaping and e-juicing at the same time” (28, M). “This is the Myblu intense. It’s the 3.6 nicotine edition one and this is the one in my hand right here. This one [device] here is an upgrade, it’s pretty strong compared to other ones that I’ve had before … just my go-to favorite because this one is just, you could put it in your pocket and it’s strong” (28, M). “[The Blu brand has] such a strong concentration zone. You actually feel like the actual nicotine’s all over your... you feel it, compared to the other cheap ones they have, they’re not as strong” (28, M).“For example, sometimes if I haven’t used this [SMOK mod] for a while and I’ve been using a pod, or if I’m using some other kind of alternative, going back to this will be the first time in a while I’ll get an actual rush. It’s like ‘oh, okay, this is what I was actually missing’” (32, M).
**Cloud size**	“The cloud isn’t a big thing. I mean, I remember whenever people first started vaping that was a big thing, the tricks and stuff” (28, M).	“That’s one of the reasons why I don’t really use JUUL stuff, because it doesn’t really produce a larger cloud and it’s also kind of satisfying to just have bigger ones, rather than just small. […] Sometimes when you just get a small one [cloud], it’s just like I don’t know, something about it just isn’t really as satisfying as having a big hit and a bigger cloud” (21, F). “I personally really like the sub-ohm mods for the reason that they do produce a lot of clouds because I can take one really big inhale and I get a lot of nicotine at once as opposed to with my little pod mod, it’s like I have to hit it several times to get the same effect as I do with just one really good hit off of this one” (29, M).
**Battery**	“Battery life is a big thing for me” (32, F).	“This one [device], I can’t tell how much battery I have left. It’s just like when it’s dead, it’s dead. There’s no indicator. Whereas this actually will show on screen how much battery I have left. Then, I know I need to change batteries and put in a new one or charge it again. That’s important to me” (35, F).
**Aesthetics**	“We just need to get something that is good-looking, in trend, stylish” (40, F).	“The main reason why I chose Blu is because it still has that cigarette feel, like when you’re holding it” (32, F). “I know that my mod has different colors and different types of designs that you can pick from. So yeah, I like to kind of color match mine” (21, F). “The Blu, I like, like she said, because it makes you feel like you’re still smoking a cigarette” (30, F).
**Ease of use**		“But this one here is a good substitute because sometimes you don’t want to carry a big vaporizer in your pocket. You just want to have something small you could put in pocket next to your cellphone” (28, M). “For me, I usually use the tank. I have a drip too, but I usually use the tank, it’s just easy. Just fill it up and go” (25, M).
**Cost**		“[Refillable tanks are] affordable, compared to disposable pods. Juul pods could be $16 to $20 and you can get that equivalent amount if you just buy a straight bottle. You have to spend like $1000 in Juul pods to equal the same amount, so it’s more cost effective to use a refillable vape” (23, M). “They sold those [Vuse Alto] for like $0.99 at the [convenience store] near my apartment” (19, F). “Don’t buy the Suorin. Look what it did to me [made my tonsils bad]. Okay, I wouldn’t buy it, unless it’s kind of cheap, so you may go buy it”(21, F).

Notes: ^a^ General to ENDS: discussion of reasons that focus on ENDS in general, without mentioning specific device type or feature. ^b^ Device-specific: discussion of reasons tied to using a specific ENDS device, brand, or feature.

## Data Availability

The data supporting this study’s findings are available on request from the corresponding author. The data are not publicly available due to containing information that could compromise the privacy of research participants.
